# Randomization in the age of platform trials: unexplored challenges and some potential solutions

**DOI:** 10.1186/s12874-025-02693-0

**Published:** 2025-11-22

**Authors:** Olga Kuznetsova, Jennifer Ross, Daniel Bodden, Freda Cooner, Jonathan Chipman, Peter Jacko, Johannes Krisam, Yuqun Abigail Luo, Tobias Mielke, David S. Robertson, Yevgen Ryeznik, Sofia S. Villar, Wenle Zhao, Oleksandr Sverdlov

**Affiliations:** 1 Merck & Co., Inc., Rahway, NJ USA; 2 Almac Group, Souderton, PA USA; 3 RWTH Aachen University, Aachen, Germany; 4 US Food and Drug Administration, Silver Spring, MD USA; 5 University of Utah School of Medicine, Salt Lake City, UT USA; 6 Berry Consultants, Abingdon, Oxfordshire, UK & Lancaster University, Lancaster, UK; 7 Boehringer-Ingelheim Pharma GmbH & Co. KG, Biberach, Germany; 8 Janssen-Cilag GmbH, Neuss, Germany; 9 MRC Biostatistics Unit, University of Cambridge, Cambridge, UK; 10 Department of Mathematics, Uppsala University, Uppsala, Sweden; 11 Medical University of South Carolina, Charleston, SC USA; 12 Novartis Pharmaceuticals Corporation, East Hanover, NJ USA

**Keywords:** Adaptive design, Platform trial, Randomization, Unequal allocation

## Abstract

While platform trials have several benefits with their adaptive features, randomization challenges become of central relevance to the design and execution of a platform trial. This paper intends to address these challenges and explore some potential solutions. A platform type of clinical trial is a clinical trial design where multiple interventions are investigated simultaneously often against partly or fully shared controls, with new treatment arms added and completed treatment arms removed. Unequal allocation is often used in platform trials to improve statistical efficiency, deliver benefits to trial participants, and control the speed of enrollment in different treatment arms. Changes to the allocation ratio may be required after an interim analysis even when the number of treatment arms remains constant, for example, in a platform trial with response-adaptive randomization. To deliver the design efficiencies promised by the carefully optimized allocation ratio or simply to ensure a pre-determined allocation ratio, randomization methods that keep allocation proportions close to the target allocation ratio throughout randomization are helpful. Other situations commonly occurring in platform trials require special considerations for randomization methods and in some cases new classes of randomization methods. Such specific platform features include the requirement to accommodate differences in eligibility for different treatments, the need to ensure partial blinding with a 2-step randomization when mode of administration for different interventions is conspicuously different and full blinding is unfeasible, the objective to balance through dynamic randomization multiple prognostic factors or the need to accommodate limited drug supplies at the numerous trial centers, among others. The key to a successful execution of a complex randomization in the platform trial is the expert design of the Interactive Response Technology (IRT) system, where the system is built at the master protocol level and existing and potential randomization needs are incorporated from the outset. An additional, often overlooked, challenge when working with unequal allocation ratios and randomization methods to attain these, is the importance of preserving the unconditional allocation ratio at every allocation. Failure to do so might lead to a selection and evaluation bias even in double-blind trials, accidental bias, and reduced power of the re-randomization test.

## Background

Conventional clinical research is an inefficient and siloed process, in which many costly and burdensome trials are conducted by different parties in parallel to address similar or highly related research questions. Modern clinical research aims to increase efficiency by investigating multiple interventions, disease or participant subtypes under common overarching master protocols.

Basket, umbrella, and platform trial designs are the three major types of master protocols [1, 2]. In the current work we focus on platform trials, as novel randomization challenges are most prevalent in them. According to a recent paper [3], a platform trial investigates “multiple targeted therapies in the context of a single disease in a perpetual manner, with therapies allowed to enter or leave the platform on the basis of a decision algorithm.” In such a context, every time an intervention is added (or removed), the question of what the new allocation ratio will be and how this should be ideally implemented through a suitable randomization method needs to be addressed.

The focus of this paper is on addressing the statistical considerations and operational challenges for implementing randomization in a platform trial. The target audience for this paper are biostatisticians and clinical investigators tasked with the design, conduct, analysis, and interpretation of a platform trial, as well as regulatory and scientific/medical journal reviewers. In this paper, we provide an overview of some important and less well known statistical and operational challenges pertaining to the randomization component of platform trials. Recognizing the breadth of the concept of randomization in this context, in this paper we focus on some concrete examples we identified as relevant. Some of our findings and recommendations may not be generalizable to more complex clinical trial settings and our work highlights areas where further efforts are needed to fill those gaps.

Platform trials offer two key efficiency advantages over running multiple separate trials in parallel. The first is operational efficiency, achieved by establishing a shared network of research centers that continuously evaluate multiple interventions using standardized protocol elements [4]. This setup can reduce the amount of trial start-up activities, lead to higher quality data, and improve feasibility of studying multiple interventions. The second is statistical efficiency, which comes from sharing a common control group. This approach can reduce the required sample size and support a more efficient decision-making, as compared to testing multiple interventions in separate trials conducted in parallel [5]. Overall, a key advantage of platform trials is their flexibility: new treatment arms can be added over time while still benefiting from the shared control group, making the trial structure more adaptive and resource-efficient.

Platform trial designs are applicable across the spectrum of clinical drug development [6]. They may particularly be useful in phase II settings where the main purpose is to screen multiple compounds and take the “most promising” ones for testing in pivotal trials [7], and in phase II/III settings which combine an exploratory phase II screening part and the phase III confirmatory part in a single protocol [8]. Platform trials are a natural extension of multi-arm multi-stage (MAMS) trials [9]. For example, the STAMPEDE trial [10, 11] was initiated as a phase II/III MAMS design in 2005 to investigate the efficacy of interventions in treating prostate cancer and was extended to a platform trial through addition of novel interventions between 2011 and 2018 [12]. Over 18 years of investigation, it randomized almost 12,000 participants and tested the efficacy of 11 prostate cancer interventions (https://www.stampedetrial.org/centres/information-on-stampede/).

The COVID-19 health emergency provided an impressive example showcasing the ability of platform trials in accelerating the research and development process and timely informing decision-making. In February 2020, the World Health Organization (WHO) published Research and Development (R&D) Blueprints for investigation of interventions in COVID-19 [13]. The R&D Blueprints served as a common building block for trial designs, thereby accelerating protocol development. Common trial design elements may also inform comparisons across trials. Thousands of trials were initiated early in the pandemic, with the aim to identify effective interventions for COVID-19 patients. Many of those trials investigated the same interventions (such as Hydroxychloroquine, Vitamin-C, Remdesivir), while many were also too small to support informed decision-making. This inefficiency was in stark contrast to several coordinated research efforts, implemented through platform trials, such as PRINCIPLE, REMAP-CAP, and RECOVERY, amongst others. For example, RECOVERY [14] is platform trial sponsored by the University of Oxford, which from March 2020 through April 2024 randomized almost 50,000 participants and successfully assessed 12 interventions, including lack of clinical benefit for Hydroxychloroquine [15] and efficacy of Dexamethasone [16] for hospitalized patients with severe respiratory complications.

The potential benefits of platform trials should be carefully weighed against challenges, which include the extra time and resources needed for trial planning, increased complexity in implementation, and some well discussed statistical challenges, such as the need for strong control of the type I error rate and blinding when interventions are of a different appearance. While the concept of platform trials is relatively new, some important lessons learned, practical guidelines, and recommendations for planning and implementing future platform trials are emerging [17–19]. The industry and academia-wide efforts to develop and extend the methodology of platform trials include the EU PEARL (EU Patient-cEntric clinicAl tRial pLatforms) (https://eu-pearl.eu) and CTTI (Clinical Trials Transformation Initiative) (https://ctti-clinicaltrials.org/our-work/novel-clinical-trial-designs/master-protocol-studies/). Several guidance documents on master protocols and platform and umbrella trials have been released by US Food and Drug Administration [20–22] and European Medicines Agency [23]. The emergence of platform trials has triggered a surge of statistical methodology research on the development of novel designs [24–27], the investigation of statistical properties of adding treatment arms to ongoing trials [28, 29], the use of historical and concurrent control data [30–32], as well as on the issues of multiplicity and type I error control [33–35].

Despite an emerging volume of work (and awareness) around the above-mentioned operational and statistical challenges for platform trials, the issues of effectively and efficiently implementing a randomization procedure when the number of treatments and the target allocation ratio among these is changing over time and when it is possibly unequal by design has received little to no attention. Randomization is an essential component of any randomized controlled trial (RCT) [36, 37]. A major strength of a randomized allocation compared to a deterministic allocation is that the former approach helps mitigate the risk of selection bias, especially in open label trials where investigators can make intelligent guesses on upcoming treatment assignments in the sequence and use this knowledge for selectively assigning trial participants to treatment arms. Also, randomized allocation helps reduce bias due to temporal effects that may arise in trials with rapid temporal change of disease and response to therapy (e.g., COVID-19 [38]) or slow recruitment such as in rare disease settings. In addition, randomization promotes the comparability of treatment arms with respect to known and unknown confounders and helps ensure unbiased causal estimates of treatment effects. Some systematic approaches have been developed for selecting an appropriate randomization method for a 1:1 RCT [39–41]. However, in platform trials, the choice of a fit-for-purpose randomization method remains an open question due to the numerous complex features of platform trial designs that will be discussed momentarily.

The importance of a careful choice of the randomization method in a platform trial is amplified by the relatively large number of platform trials utilizing response-adaptive randomization (RAR) [42, 43]. In situations of competitive enrollment, RAR may result in prioritized enrolment to treatment arms which are deemed more efficacious, thereby accelerating research. However, RAR encompasses a wide range of different designs for which additional considerations are needed to avoid increasing sources of bias. See Robertson et al. [44] for an in-depth discussion of issues linked to trial designs using RAR.

From a statistical perspective, the determination of a target allocation ratio has been discussed in Viele [45] and Bofill Roig et al. [46], suggesting that optimal allocation ratios in platform trials require adapting the allocation rules and/or using unequal allocation ratios such as Dunnett’s optimal allocation based on statistical efficiency consideration recommended in the FDA draft guidance on Master Protocols for Drug and Biological Product Development [22]. However, how to randomize trial participants in a specified allocation ratio is an important open question, as the choice of a randomization method impacts statistical properties of the implemented design and analysis. Throughout this paper, we will assume that an (adaptive) allocation rule for determining the target allocation ratio is given. Therefore, our focus will be not on ways for determining such ratios, but rather on the methodologies for actual random assignment of participants to the interventions within a platform trial according to the specified allocation ratios.

Frequently, clinical trial protocols and papers reporting platform trial results provide very little details on how randomization for the chosen allocation ratio was implemented – e.g., using multinomial probabilities, blocks, etc. as well as on why this implementation was selected. While the details of the implementation of the randomization are typically avoided in study protocols to reduce the potential for selection bias on behalf of investigators, the details are typically specified in internal documents (for example, interactive response technology (IRT) specifications) and should be included in the publications reporting study results. Some randomization methods may be more fit-for-purpose than others, and the knowledge of all details on the implemented randomization methodology in a platform trial is paramount for the integrity, credibility and interpretation of trial results. 

Tables 1 and 2 provide a Glossary of some important terms relevant to platform trials that will be used throughout the paper. The “Methodological challenges” section describes some important statistical issues that stem from the choice of a randomization method in a platform trial. The “Operational considerations” section describes some important practical considerations for implementing the chosen randomization method using an IRT, including added complexity of the study blinding. The “Conclusions” section provides a summary of the identified challenges, possible solutions, open problems, and the motivation for future research.

**Table 1:** Glossary of platform trials.

Term used in this paper	Alternative terms or specific examples used in the literature	Description
Platform trial (or briefly trial)	Adaptive platform trial; Master protocol trial; Platform study	A clinical trial governed by a master protocol allowing for one or more treatment arms to be added via intervention-specific appendices to the master protocol during the course of the trial.
Umbrella trial	Umbrella study	A clinical trial governed by a master protocol where all treatment arms start at the same time.
Intervention	Treatment; Procedure; Drug	A treatment or procedure applied to the clinical trial participants.
Investigational intervention	Experimental intervention	An intervention whose efficacy and or safety is investigated in the clinical trial.
Control intervention	Control; Comparator intervention	An intervention whose efficacy and or safety is used as a comparison benchmark for investigational intervention.
Treatment arm	Study arm; Arm; Treatment group	A set of participants allocated (or randomized) to the same intervention.
Investigational arm	Experimental arm	A set of participants allocated (or randomized) to the investigational intervention.
Control arm	Comparator arm	A set of participants allocated (or randomized) to the control intervention.
Treatment kit	Kit; Drug kit; Medication; Pack	A medication package to be dispensed to a participant according to their treatment arm/study visit, typically labeled with a coded identifier of the contents (masked in double-blind studies).
Shared control	Shared control arm; Shared control group	A control arm used in more than one comparison with an investigational arm.
Intervention-specific appendix	Substudy; Subgroup; Arm	A set of one or more treatment arms that join the platform trial together as needed for investigating an intervention, including intervention-specific inclusion/exclusion criteria.
Pooled control		The data from the intervention-specific control arms pooled together for the analysis. The master protocol and/or each intervention-specific appendix may specify the conditions (e.g. temporal, co-randomized, subpopulation, etc.) to be satisfied by participants to be included in each intervention-specific pooled control.
Arm opened	Arm addition; Arm entering; Arm inclusion; Arm introduction; Active arm; New arm	The (temporary) state of an arm meaning that it can be considered for allocation of an eligible participant at a given center.
Arm closed	Arm dropped; Arm retired; Arm discontinued; Arm exited	The (temporary or permanent) state of an arm meaning that it cannot be considered for allocation at a given center. An arm can be closed for a variety of reasons, e.g. permanently closed due to legal or administrative restrictions at the center, due to reaching the planned final number of allocated participants, for futility, for efficacy, for safety - all at the trial level -, or temporarily closed due to reaching the planned interim number of allocated participants in the trial or at the center or for particular subpopulation, for unavailability of treatment kits or staff at the center, etc.
Participant	Patient; Subject	A member of the population enrolled in the master protocol.
Subpopulation	Participant type; Subgroup; Eligibility group	A subset of the population.
Center	Site	A medical research institution where participants can be enrolled to the platform trial, which may restrict eligibility of certain arms due to restrictions at a higher (e.g., country/region) level.
1-step randomization	1-level randomization; 1-tier randomization	The randomization performed directly among the treatment arms the participant is eligible for.
2-step randomization	2-level randomization; 2-tier randomization	The randomization to one of the investigational arms or its matching control performed in two steps: (1) randomization to the investigational arm/matching control pair; (2) randomization to the investigational arm vs. matching control within the pair identified in step 1.

**Table 2:** Glossary specific to randomization methods

Term used in this paper	Alternative terms used in the literature	Description
Randomization method	Randomization procedure; Allocation method; Randomization scheme	A theoretical method that prescribes how to assign participants to the randomization units (for example, treatment arms).
Target allocation ratios vector	Target allocation ratio; Randomization ratio; Allocation ratio; Intended allocation ratio;	A vector of non-negative real numbers, indicating the ratios of participants that should be allocated to each randomization unit (for example, treatment arm), typically separated by “ :”. For example, 1.5:1:1:1 or sqrt(3):1:1:1.
Fixed randomization	Fixed randomization method	Randomization scheme where the randomization rule does not depend on accumulating covariate, outcome or other data from the trial; the randomization sequence (schedule) can be generated prior to the start of randomization.
Dynamic allocation	Adaptive randomization	Randomization method that requires input based on covariates, outcomes, or any other information obtained after the start of randomization, to generate the treatment assignment for the next participant; the randomization sequence cannot be generated in advance.
Randomization schedule	Randomization sequence; Randomization list; Allocation schedule; Allocation sequence	The sequence of treatment arm assignments to be given to the participants in order of randomization. The sequence is generated following the fixed randomization method chosen for the trial.
Complete randomization	Simple randomization	Randomization where each participant is randomized independently in a given allocation ratio.
Restricted randomization		Randomization where the treatment assignments of the participants are not independent.
Stratified randomization		Randomization where a population is broken into non-overlapping subpopulations and independent randomization schedules are prepared for each subpopulation.
Multiple-dummy blinding		Blinding in a $\:K$-arm trial where a participant in an intervention arm receives one intervention and $\:(K-1)$ controls matching in appearance other interventions and a participant in a control arm receives $\:K$ controls.
Real-time adaptation		An implementation approach to handling pre-specified modification to a trial’s design or conduct. The types of modifications (e.g., open/close treatment arms, ratio changes) and how they are triggered (e.g., formal interim analysis of accumulating data, occurrence of adverse events, expanding study needs) are planned and specified within the protocol. This approach allows modification to be applied to the trial design in real-time.

## Methodological challenges

Platform trial designs are multi-center, multi-arm (adaptive) designs with dynamically changing allocation ratios driven by opening and/or closing of treatment arms. The arms can be closed temporarily or permanently for superiority, futility, safety, or other reasons. Each of these characteristics on its own creates randomization challenges. In platform trials, all these characteristics may occur at the same time increasing complexity of randomization and requiring close evaluation at design stage.

Unequal allocation procedures, often required in platform trials, need to preserve the unconditional allocation ratio at every allocation. Additionally, since interim analyses are often based on small sets of participants of unknown size, the allocation procedure needs to provide a tight adherence to the target allocation ratio throughout randomization. The imbalance in important prognostic factors often investigated in platform trials can lead to biased results, and thus stratification (by a small number of prognostic factors) or dynamic allocation (when there are many prognostic factors) might need to be incorporated in a randomization schedule to provide treatment arms that are similar in prognostic factors [47]. Differences in eligibility criteria across the treatment arms require special allocation techniques. Central randomization to several treatment arms might make maintaining sufficient drug supplies at numerous study centers problematic and require dynamic allocation for economical drug use, often necessary in early stages of drug development.

Inability to mask the treatment by giving all treatments the same appearance or by multiple-dummy blinding, where a participant receives one active treatment and matching placebo for all other treatments (or matching placebo for all treatments if the participant is assigned to the placebo arm) leads to a partial blinding. It is often executed through a 2-step randomization, where each investigational treatment has its own matching control. During the 1^st^ step participants are randomized to an investigational treatment/matching control pair that is not masked; during the 2^nd^ step, participants are randomized to an investigational treatment vs. matching control within a pair in a masked manner. When appropriate, the matching control arms are combined into a pooled control arm for analysis. A 2-step randomization might also be implemented in different settings, for reasons other than the partial blinding, for example, where the 1^st^ step randomizes participants to a substudy, and the 2^nd^ step randomizes participants to one of the treatment arms within a substudy.

In what follows, we discuss these statistical challenges in detail.

### Preserving the unconditional allocation ratio at every allocation step with unequal allocation randomization procedures

The *unconditional* allocation ratio for a given participant in the sequence is the allocation ratio in which this participant will be randomized without conditioning on the current treatment arm totals (that is, the number of participants in each treatment arm), whereas the *conditional* allocation ratio is one that is conditioned on the current treatment arm totals. Since platform trials often utilize unequal allocation designs across segments of randomization, the requirement for the allocation ratio preserving (ARP) property is explained here for further awareness when choosing an existing or developing a novel unequal randomization technique. In platform trials, the decisions to add or remove the treatment arms and change the allocation ratio for the next segment of randomization can be based on the assessment of some available trial information, including the current treatment arm totals. After the unequal allocation ratio is determined and specified for the next segment of randomization, the unconditional allocation ratio should be preserved across that segment.

The idea that allocation procedures should be expanded to unequal allocation in a way that preserves the unconditional allocation ratio at every allocation step originated in the 2011–2012 research by Kuznetsova and Tymofyeyev [48–50] but has not yet spread wide into the statistical community. For equal allocation procedures symmetric with respect to the treatment arms, the ARP property is automatically preserved – due to symmetry, all participants are randomized in equal unconditional allocation ratio regardless of their allocation order. However, this is not necessarily the case with unequal allocation procedures.

Consider a trial with $\:{r}_{1}:{r}_{2}$ allocation to Treatment 1 and Treatment 2, where $\:{r}_{1}\ne\:{r}_{2}$ are arbitrary positive numbers. The allocation ratio can also be expressed through the allocation proportions $\:{\rho\:}_{1}$ and $\:{\rho\:}_{2}$, where $\:{\rho\:}_{1}=\frac{{r}_{1}}{{r}_{1}+{r}_{2}}$, $\:{\rho\:}_{2}=\frac{{r}_{2}}{{r}_{1}+{r}_{2}}$, and thus $\:{\rho\:}_{i}\in\:\left(\mathrm{0,1}\right)$, $\:{\rho\:}_{1}+{\rho\:}_{2}=1.$ Denote by $\:{N}_{i1}$ and $\:{N}_{i2}$ the treatment arm totals after the $\:i$-th allocation. Variations in the unconditional allocation ratio commonly arise in a trial where the preferred treatment at the next allocation is a function of the difference in the treatment arm totals divided by the target allocation proportions, $\:|{N}_{i2}/{\rho\:}_{2}-{N}_{i1}/{\rho\:}_{1}|$, as is the case with the unequal allocation expansion of the biased coin randomization and minimization by Han et al. [51].

**Fig. 1:**
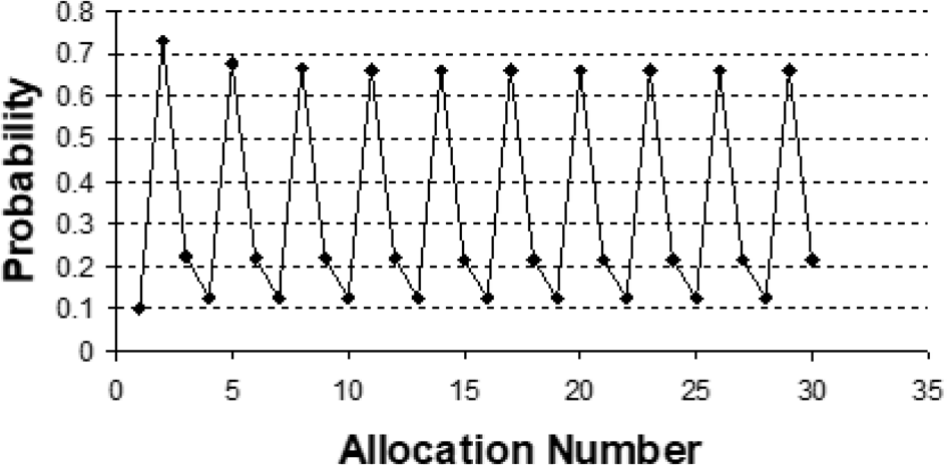
Unconditional allocation probabilities to Treatment 1 with 1:2 biased coin minimization allocation by Han et al. [51] to Treatment 1 and Treatment 2 that balances only on treatment arm totals

Figure 1 displays the unconditional probabilities of Treatment 1 allocation with the Han et al. [51] 1:2 biased coin randomization to Treatment 1 and Treatment 2 derived by Kuznetsova and Tymofyeyev in [50]. The unconditional probability of Treatment 1 assignment is low (0.1 to 0.2) at allocation steps 1, 3, 4, 6, 7, 9, etc., while this probability is very high at allocation steps 2, 5, 8, etc.

Variations in the unconditional allocation ratio are undesirable for several reasons [50]. They provide potential for accidental bias, especially in multi-center trials with randomization stratified by center. If the first participant in a center has a better prognosis than the participants randomized later, and if there is higher than average probability of randomizing this participant to Treatment 2 (as in the example above), the accidental bias will be added up across the centers.

Such variations also provide potential for selection bias (described earlier) and evaluation bias (where the investigator evaluates the outcomes while being aware of the treatment assignment) even in double-blind trials, where selection bias does not occur when the unconditional allocation ratio is preserved [50]. Indeed, if in a single-center trial the investigator knows that (as in the example above), the 1^st^, 3^rd^, 4^th^, 6^th^, etc., participants have lower than average probability of being assigned to Treatment 1 (the control), they may randomize participants with better prognosis at these allocation steps and randomize the participants with the worse prognosis at other allocation steps, where the probability of allocation to Treatment 1 is higher than average. This will make the Treatment 1 arm and Treatment 2 arm incomparable in prognosis at baseline and will likely bias the results in favor of Treatment 2 arm. Thus, the investigator will not even need to be unblinded to the past treatment assignments to try to guess the next treatment assignment and introduce selection bias in a trial with variations in the unconditional allocation ratio. The evaluation bias can also be introduced in a double-blind trial by the knowledge of variations in the unconditional allocation ratio.

Variations in the unconditional allocation ratio also cause a shift in the distribution of the randomization test statistics which lowers the power of the re-randomization test [48–50, 52]. In the example of a trial with a randomization method proposed by Han et al. [51], simulated by Kuznetsova and Tymofyeyev in [50], the power of the randomization test was reduced from 90% to 80%. Additionally, as shown by Kaiser in [53], such variations cause the treatment effect estimator to be biased from a randomization-based inference perspective. A randomization test is often required by regulatory authorities in trials with dynamic allocation; it also, under very general conditions, preserves the Type I error with RAR often used in platform trials [54].

The greater the variations in the unconditional allocation ratio, the greater are the potential biases and the reduction in the power of the re-randomization test [50]. Typically, a greater forcing towards the target allocation ratio in non-ARP procedures – for example, a higher value of the biased coin probability in the biased coin randomization by Han et al. [51] – results in higher variations in the unconditional allocation ratio. Thus, if one must use a non-ARP procedure, a trade-off between the closeness to the target allocation ratio and the consequences of the variations in the unconditional allocation ratio should be carefully considered as this can impact the potential for bias as well as efficiency of some statistical/inferential approaches.

The ARP problem is also prominent with the expansions of the covariate-adaptive randomization procedures to unequal allocation. For a dynamic covariate-adaptive randomization [37, 55, 56], the ARP property means that for the sequence of covariates observed in the trial, the unconditional allocation ratio is the same at every allocation step. Since the sequence of covariates to be observed in the trial is not known in advance, a more general requirement is the preservation of the unconditional allocation ratio for any sequence of covariates.

One simple solution to ensure the ARP property with unequal allocation in a ratio that can be expressed through integers is an approach called “mapping” [50]. It could be explained on the example of generating the permuted block randomization (PBR) [57] to the treatment arms A, B, and C in 1:2:3 ratio. Following the mapping approach, one would first generate the equal allocation PBR to 6 “fake” treatment arms. Then the first “fake” treatment arm will become the actual treatment arm A, pooled “fake” treatment arms 2 and 3 will form the actual treatment arm B, and pooled “fake” treatment arms 4, 5, and 6 will form the actual treatment arm C.

This approach can be described formally for an arbitrary allocation procedure in the following way. Suppose an allocation procedure defined for equal allocation to any number of treatment arms needs to be expanded to allow allocation to $\:K\:\ge\:\:2$ treatment arms $\:{G}_{j}$ ($\:j\:=1,\dots\:,K$) in $\:{Q}_{1}:{Q}_{2}:\:\dots\:\::{Q}_{K}$ ratio, where $\:{Q}_{1},{Q}_{2},\dots\:,{Q}_{K}$ are integers with the greatest common divisor of 1 and $\:S\:={Q}_{1}+{Q}_{2}+\dots\:+{Q}_{K}$ (we will call *S* the block size). First, an equal allocation to $\:S$ “fake” treatment arms $\:{F}_{1},\:{F}_{2},\:\dots\:,\:{F}_{S}$ is executed following the algorithm defined for equal allocation to $\:S$ treatment arms. Then the first $\:{Q}_{1}$ “fake” treatment arms are mapped to treatment arm $\:{G}_{1}$; the next $\:{Q}_{2}$ “fake” treatment arms are mapped to treatment arm $\:{G}_{2}$; …; and finally, the last $\:{Q}_{K}$ “fake” treatment arms are mapped to treatment arm $\:{G}_{K}$. Due to symmetry with respect to the “fake” treatment arms, such procedure will provide equal unconditional allocation to $\:S$ “fake” treatment arms $\:{F}_{1},\:{F}_{2},\:\dots\:,\:{F}_{S}$ at every allocation. Thus, it would provide unconditional $\:{Q}_{1}:{Q}_{2}:\:\dots\:\::{Q}_{K}$ allocation ratio to treatment arms $\:{G}_{j}$, where $\:j\:=1,\dots\:,K$, at every allocation.

Other examples of ARP procedures obtained through mapping include block urn design [58] and drop-the-loser urn design applied to fixed unequal allocation [59, 60]. The mapping approach was applied to expand various equal allocation procedures to unequal allocation [49, 50, 61–63].

While mapping works well when the block size $\:S$ is small, as is the case for 1:2, 1:3, or 2:3 allocation ratios common in clinical trials, with the large block size, like 5:5:7 allocation, the adherence to the target allocation ratio in small, randomized sets of participants might be suboptimal. Indeed, the allocation space for such procedures is at least as wide as the permuted block space with the permuted block size *S*. In the latter case, other ARP approaches are needed to keep the allocation ratio close to the target one throughout the randomization; however, only a handful of ARP unequal allocation procedures not based on expansion through mapping were developed so far. Kuznetsova and Tymofyeyev [62, 64] expanded the 2-arm brick tunnel randomization (to be described in the next section) to generate an $\:{Q}_{1}:{Q}_{2}$ ARP allocation (wide brick tunnel) that allows all allocation sequences with maximum tolerated imbalance $\:MTI\le\:b$, where $\:MTI={N}_{2}/{Q}_{2}-{N}_{1}/{Q}_{1}$. Kuznetsova and Plamadeala Johnson [65] offered several ways to expand the 2-arm equal allocation biased coin design [66] to a 2-arm ARP unequal allocation.

Of note, the following unequal allocation procedures do not have the ARP property: a randomized urn design [37], an expansion of the maximal procedure [67], a biased coin randomization and minimization expansion by Han et al. [51], a doubly adaptive biased coin design (DBCD) applied to fixed unequal allocation [60, 68], an adaptation of the biased coin randomization [69], a generalized method for adaptive randomization [70], the mass weighted urn design [71], amongst others.

In summary, when expanding the unequal allocation randomization tools to fit the needs of a platform trial, it is recommended to choose an ARP procedure when possible. When a non-ARP procedure is used in absence of the ARP alternatives that fit the study needs, a trade-off between the closeness to the target allocation ratio and the extent of the potential implications of a non-ARP procedure should be considered when specifying the parameters of the procedure.

### Maintaining the tight adherence to the target allocation ratio throughout the randomization

Platform trials typically have multiple treatment arms and points at which treatment arms might be added, closed, or require the change to the allocation ratio. The addition of treatment arms to a platform trial is a relevant change for which the randomization system should be prepared for at the conception of the platform trial. However, it may be an infrequent change, i.e., there may be many participants recruited between the additions of treatment arms.

The closure of randomization to treatment arms within a platform due to interim decisions, in general, should follow the same considerations as for more standard trial designs. An important difference is that interim analyses (IAs) may take place more frequently across the platform trial – depending on the operational setup and particularly so when the platform is adaptive. If IAs are triggered based on reaching certain information per intervention tested, this may lead to frequent analyses and potentially decisions across the whole platform. A decision to close treatment arms may or may not require adjustments to the target allocation ratio of the subsequent participants. Often due to the large number of interim analyses, the number of participants randomized between those analyses and design adjustments may be relatively small and often not known in advance.

Testing many treatments simultaneously will result in competitive enrollment, which could potentially slow down the evaluation of promising interventions. To mitigate this concern, many platform trials implement RAR to accelerate assessment of empirically “best” interventions. RAR requires frequent updates to randomization probabilities to work efficiently and robustly. The resulting target allocation proportions with RAR are frequently irrational-valued numbers that, in order to use permuted block randomization common in clinical trials [57], can be represented only approximately with the blocks of small sizes [72]. Increasing the permuted block size mitigates the approximation error but increases the chance of deviating from the target allocation ratio, as the participant recruitment between the frequent allocation updates can happen to be smaller than the block size.

Overall, randomization methods for platform trials need to handle unequal allocation ratios with frequent allocation updates in situations with potentially small numbers of recruited participants between the updates. Thus, if the allocation sequences do not closely adhere to the target allocation ratio, the achieved allocation ratio in a small sample can considerably deviate from the target ratio. An additional problem is that implementation of an updated allocation ratio may come with a new, independently generated, randomization schedule, which may by chance introduce large gaps between two allocations to some of the treatment arms, especially those with smaller allocation probabilities, when the two randomization schedules are combined. This highlights the importance of having a tight adherence to the target allocation ratio in each randomization schedule that might be used throughout the trial.

Additionally, a tight adherence to the target allocation ratio throughout the randomization reduces the potential for accidental bias associated with the time trend [40]. All these reasons call for randomization methods which maintain a tight adherence to the target allocation ratio throughout the randomization.

#### Using brick tunnel randomization for tight adherence to the target allocation ratio

The 2023 FDA guidance [22] describes the square-root allocation ratio $\:1:1:\dots\::1:\sqrt{K}$ (with $\:\sqrt{K}$ allocation to the control arm) as an example for increasing joint statistical efficiency (power) of $\:K$ pairwise treatment comparisons to a common control in certain settings. However, the guidance does not say how such a randomization schedule can be generated (i.e., which randomization method should be used to attain this ratio). If participants are randomized independently to ($\:K+1$) treatment arms in a $\:1:1:\dots\::1:\sqrt{K}$ ratio using complete randomization (i.e. each participant is randomly assigned with fixed allocation probability), the observed allocation proportions may substantially differ from the target allocation proportions (see Example 1 below). The allocation ratio can be approximated with integers: for example, for $\:K=2$, $\:\sqrt{2}\approx\:1.4$ and $\:1:1:\sqrt{2}$ ratio can be approximated with 5:5:7 creating a block size of 17. This ratio can now be implemented with the permuted block randomization. However, with the block size of 17, the observed allocation ratio can still deviate at any “interim look” within the block severely from the target ratio, which is achieved at the end of a permuted block. A less accurate approximation would be $\:\sqrt{2}\approx\:1.5$ with $\:1:1:\sqrt{2}$ ratio approximated by $\:2:2:3$ creating a block size of 7, whose implementation may be less likely to lead to large deviations.

To keep a tight adherence to the target allocation ratio throughout the randomization with the allocation ratios that, when approximated through integers result in a large block size (so that the PBR does not guarantee good adherence to the target allocation ratio), Kuznetsova and Tymofyeyev [48] proposed the brick tunnel randomization (BTR). The BTR is defined for allocation to $\:K\ge\:2$ treatment arms with $\:{r}_{1}:{r}_{2}:\dots\::{r}_{K}$ allocation ratio, where $\:{r}_{j}$’s could be any positive real (rational or irrational) numbers, such as $\:\sqrt{2}$ in the previous example. Using these notations, let $\:{\rho\:}_j=r_j/{\displaystyle\overset K{\underset{k=1}{\sum\;}}}r_k$ denote the target allocation proportion for the $\:j$-th treatment.

A sequence of allocations to $\:K$ treatment arms denoted Treatment $\:1$, …, Treatment $\:K$ can be visualized on a $\:K$-dimensional unitary grid, where the axes correspond to Treatment $\:1$, …, Treatment $\:K$, and one step along the $\:j$-th axis corresponds to the allocation to Treatment $\:j=1,\dots\:,K$.

##### Example 1: implementing a 5:5:7 BTR (that approximates a $\:1:1:\sqrt{2}$ ratio)

**Fig. 2:**
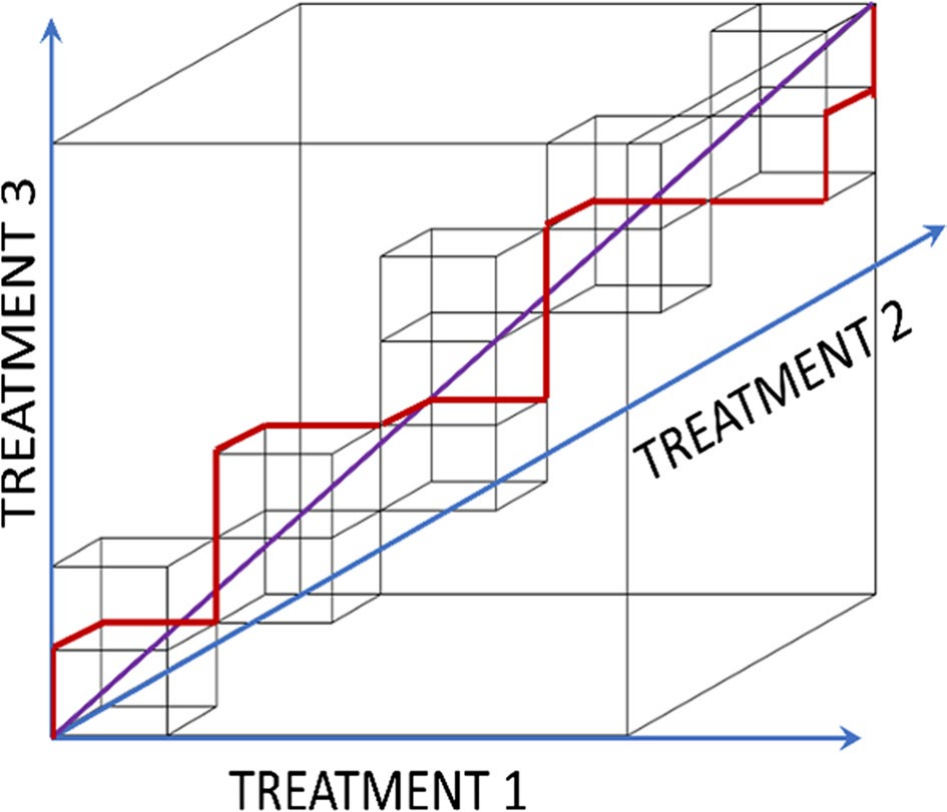
The allocation space for the brick tunnel randomization (BTR) to Treatments 1, 2, and 3 in 5:5:7 allocation ratio, pictured within the 5:5:7 block and an example of the allocation sequence

Figure 2 presents an example of the allocation sequence for the 5:5:7 BTR on the three-dimensional unitary grid. It also shows the allocation space of the BTR within the permuted block randomization allocation space for comparison. Instead of occupying the whole block of $\:5\:\times\:\:5\:\times\:\:7$ as is the case with the PBR, allocation sequences for BTR are constrained to a chain of unitary cubes pierced by the allocation ray $\:(5u,\:5u,\:7u)$ – the diagonal of the block that represents the target allocation ratio. As a result, all allocation sequences stay very close to the allocation ray and any small segment of the allocation sequence will have an allocation ratio that is reasonably close to the target ratio (which might not be the case with the PBR).

BTR not only ensures that the observed allocation ratio $\:{N}_{i1}:{N}_{i2}:\dots\::{N}_{iK}$, where $\:{N}_{ij}$, $\:j=1,\dots\:,K$ are the treatment arm totals after $\:i$ allocations, closely approximates the target allocation ratio, but that the deviation of the treatment arm totals from the “ideal” treatment arm totals $\:i{\rho\:}_{j}$ (achievable only when the “ideal” treatment arm totals are integers) is small. By design, BTR ensures that $\:\left|{N}_{ij}-i{\rho\:}_{j}\right|<1$ for every $\:i\ge\:1$. Also, by design, for BTR the conditional randomization probabilities (i.e., the probabilities of Treatment $\:j$ allocation, $\:j=1,\dots\:,K$, for the next participant given current treatment arm totals) are derived in a way that ensures the ARP property discussed above.

To illustrate the closer adherence of the BTR treatment arm totals to the “ideal” treatment arm totals compared to PBR and complete randomization (CR), we will use the Euclidean distance as a measure of imbalance between the treatment arm totals $\:{N}_{ij}$ and the “ideal” treatment arm totals $\:i{\rho\:}_{j}$, $\:j=1,\dots\:,K$ after $\:i$ allocations: $\:Imb_i=\sqrt{\sum\limits_{j=1}^K\left(N_{ij}-i{\rho\:}_j\right)^2}$ [73]. For example, with 5:5:7 BTR, after 10 participants are randomized, only 3 combinations of treatment arm totals, the closest to the “ideal” treatment arm totals (50/17, 50/17, 70/17), are possible: (3,3,4) with probability 0.88, (3,2,5) and (2,3,5) – each with probability 0.06 (so 0.12 in total for these two triplets of treatment arm totals). By contrast, the probabilities to result in these treatment arm totals, are 0.18, 0.11, 0.11 (only 0.40 in total) with PBR and 0.08, 0.07, 0.07 (only 0.21 in total) with CR. Thus, for PBR and CR the treatment arm totals are more likely to deviate more from the “ideal” treatment arm totals and thus are likely to result in the observed allocation ratio farther away from the target allocation ratio.

**Fig. 3:**
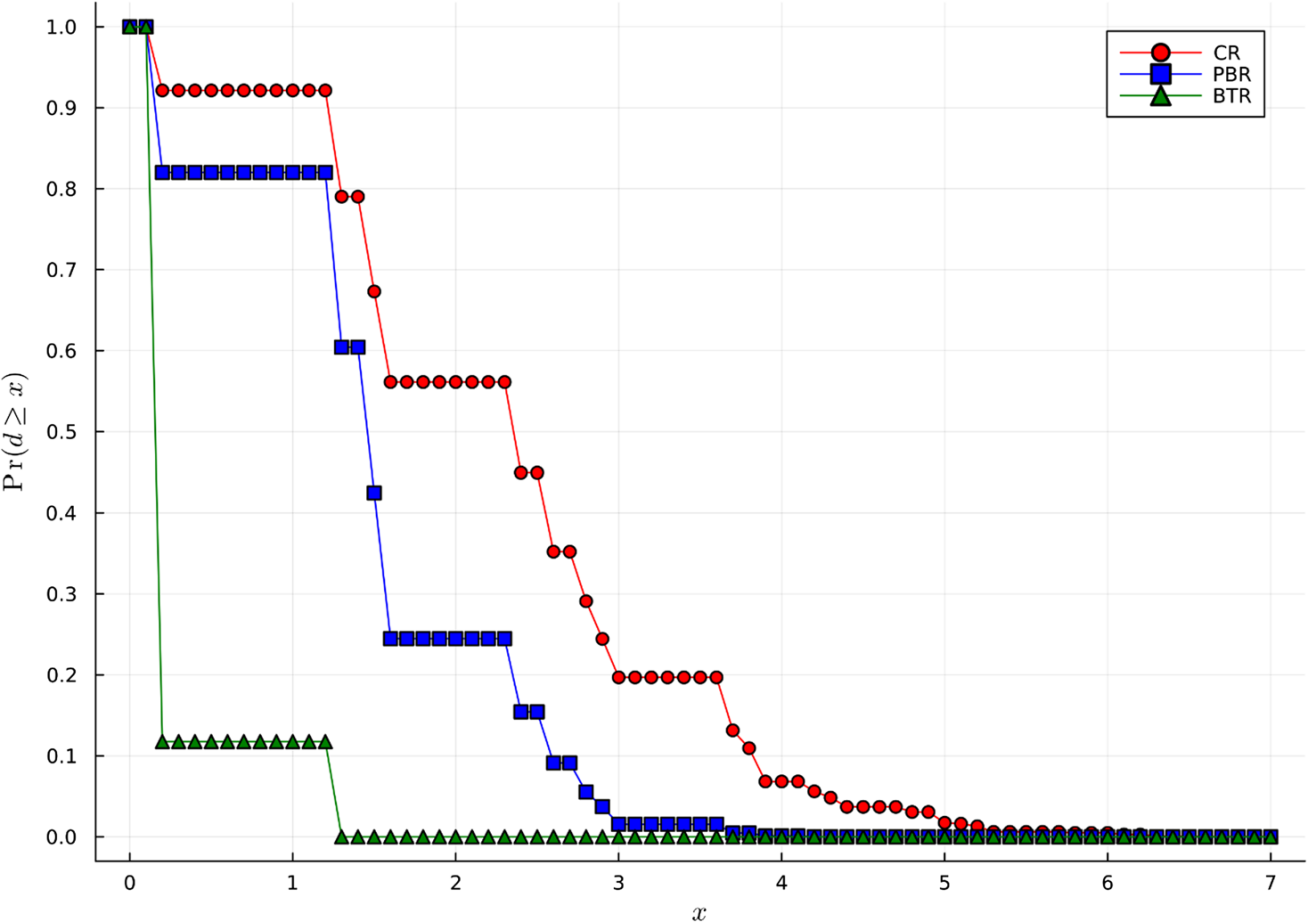
Probability that the imbalance in treatment assignments exceeds *x* with 5:5:7 brick tunnel randomization (BTR), permuted block randomization (PBR), and complete randomization (CR)

Figure 3 provides a full comparison of the distribution of the imbalance in treatment assignment after 10 participants are randomized using BTR, PBR, and CR. One can see that PBR and CR are likely to result in a considerably higher imbalance compared to BTR for a small set of 10 participants.

**Fig. 4:**
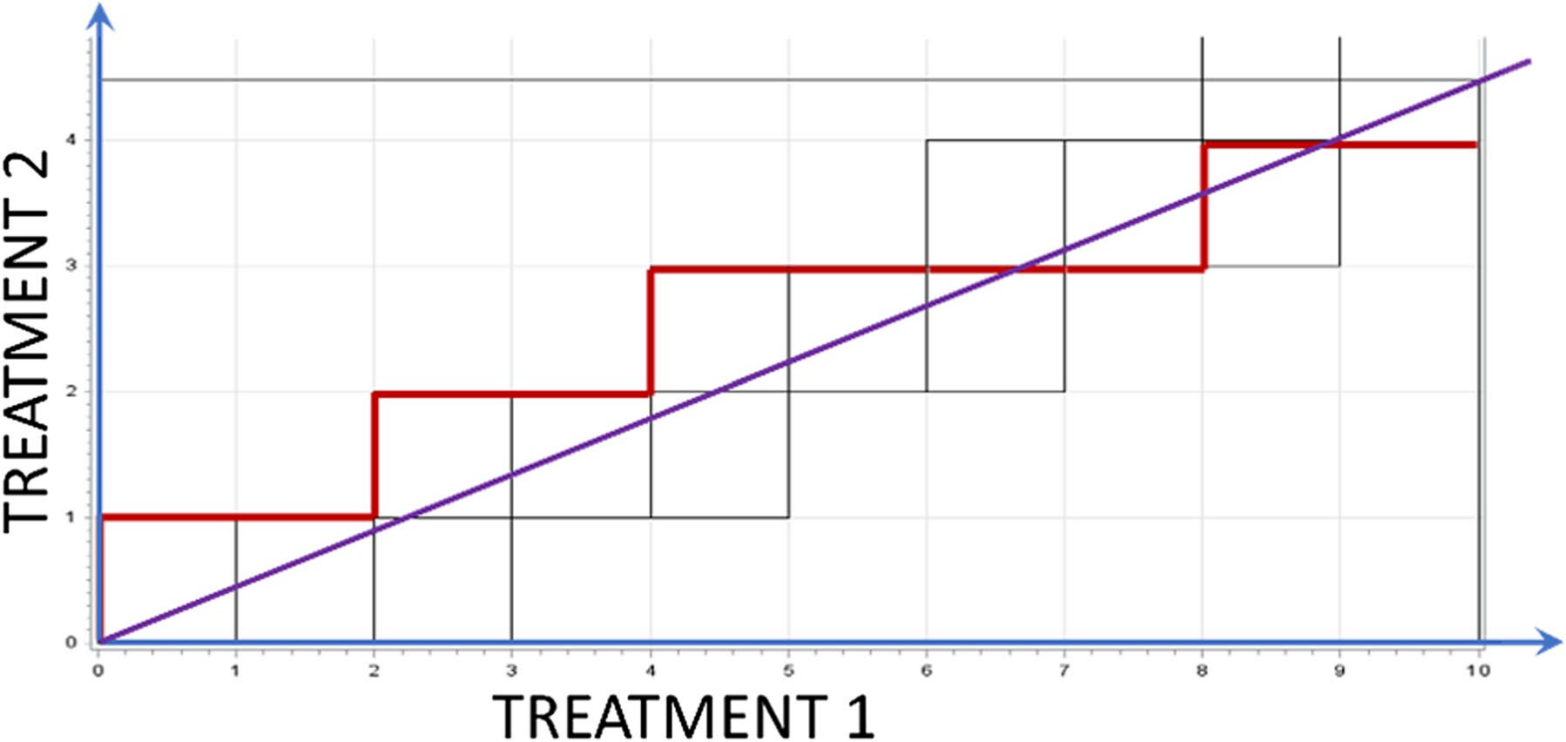
The allocation space for the brick tunnel randomization (BTR) to Treatments 1 (Pooled Treatment arms) and 2 (Control arm) in a 5:√5 allocation ratio and an example of its allocation sequence on the 2-dimensional unitary grid

To generate a BTR randomization sequence for randomization to $\:K\ge\:2$ investigational treatment arms and a control treatment arm in a $\:1:1:\dots\::1:\sqrt{K}$ ratio as in the examples considered in [22], one can first generate a 2-arm BTR sequence with $\:K:\sqrt{K}$ ratio to “Pooled Treatment arms” vs. Control arm and then replace the “Pooled Treatment arms” allocations with the PBR sequence for $\:K$ treatments with the permuted block size $\:K$ [74]. In the example of $K=5$, the 1^st^ step is the generation of the BTR with $\:5:\sqrt{5}$ allocation ratio to “Pooled Treatment arms” vs. Control arm. Fig. 4 presents the allocation space for such BTR and an example of its allocation sequence on the 2-dimensional unitary grid.

If a 2-step randomization is required in the same setting, for a $\:1:\sqrt{K}$ allocation ratio for each investigational treatment arm relative to the pooled matching controls, the 1^st^ step represents equal randomization to $\:K$ investigational treatment/matching control pairs and the 2^nd^ step represents $\:\sqrt{K}:1$ randomization to investigational treatment vs. control within each pair. The 2^nd^ step can be executed using BTR if the integer approximation of the $\:\sqrt{K}:1$ ratio results in a large block size.

BTR can be also useful in many other randomization adaptations in the platform trials.

##### Example 2: adding a treatment arm during the randomization

**Fig. 5:**
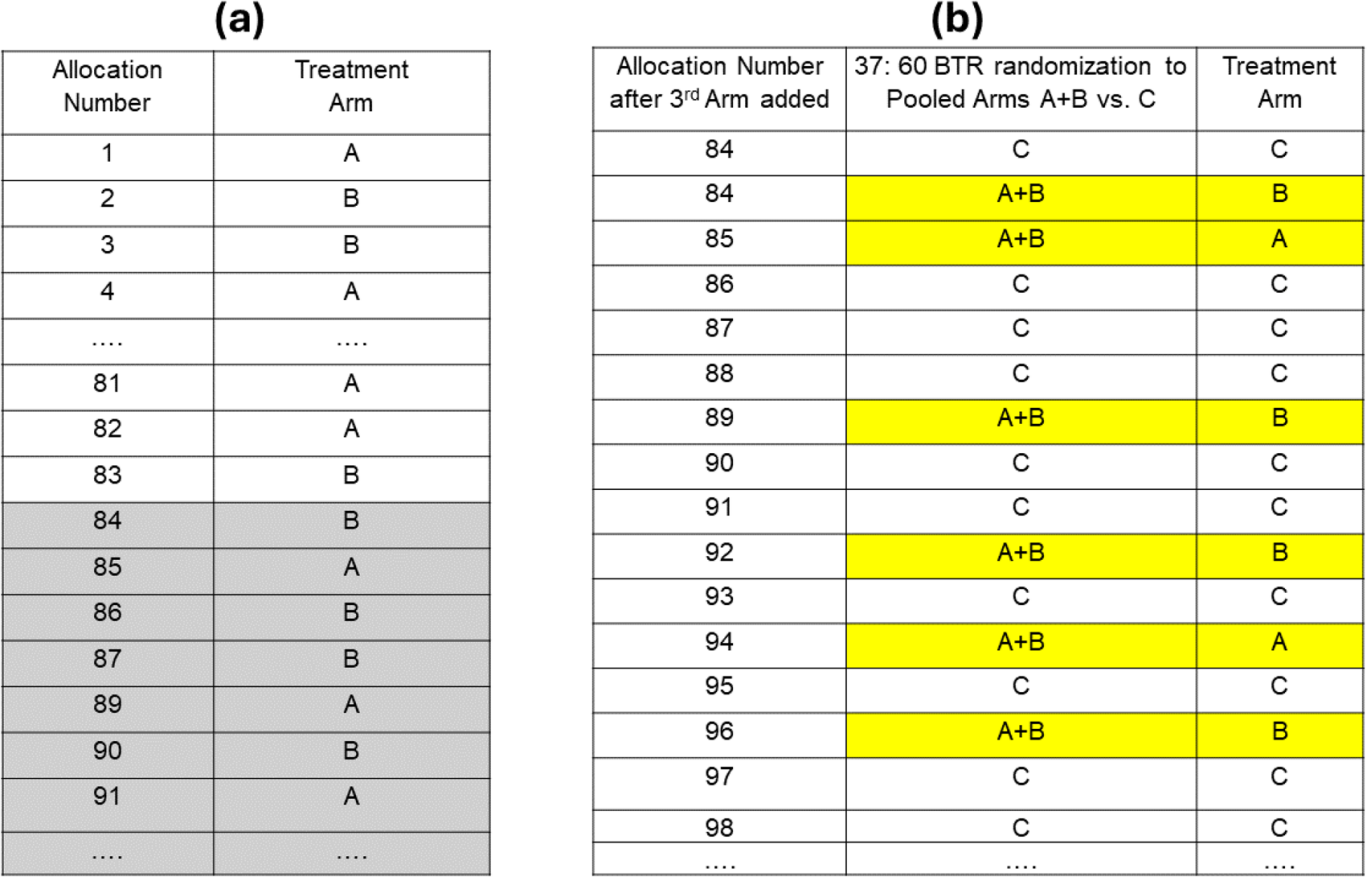
Example of adding treatment arm C to existing randomization schedule for treatment arms A and B. (**a)** Randomization schedule for original treatment arms A and B. Shaded part shows 37 allocations remained to be assigned to treatment arms A and B when treatment arm C joins the randomization. (**b)** Randomization schedule after treatment arm C joined randomization. 2^nd^ column shows the 37:60 BTR randomization schedule to pooled treatment arms A + B vs. C; 3^rd^ column shows the final schedule with the slots for A + B filled with A or B following the remaining allocations on the original randomization schedule to treatment arms A and B

Consider a platform trial that starts with 2 treatment arms, A and B, that should enroll 60 participants each (a total of 120 participants). A 1:1 permuted block schedule with the block size 4 is prepared for this randomization (Fig. 5a). A treatment arm C of 60 participants is added to randomization when 83 participants are already randomized into treatment arms A and B and thus, 37 participants remain to be allocated to these two treatment arms. How could we add 60 treatment arm C participants to the existing randomization schedule for treatment arms A and B to have them equally spread throughout the remainder of the randomization?

Figure 5b illustrates one possible solution. A 2-arm BTR sequence with 37:60 allocation to pooled treatment arms A + B vs. treatment arm C is generated (Fig. 5b, 2^nd^ column). The 37 A + B allocation slots are then filled with the existing randomization schedule for the remaining allocations to A and B (Fig. 5a, shaded assignments in the second column). Thus, the treatment arm C allocations are evenly spread across the remaining allocations to the three treatment arms. Continuing to use the original randomization schedule for treatment arms A and B preserves the balance in the treatment arm totals in these two treatment arms better than when starting a completely new schedule. This might be especially helpful with stratified randomization that otherwise results in one interrupted randomization schedule for each stratum with imbalances added across strata.

The SAS code for 2-arm and 3-arm BTR randomization, where the conditional allocation probabilities are derived explicitly [48, 74], is available at the website of the book [75]. An R package that implements BTR for 2, 3, or more treatment arms is being prepared for release to the statistical community.

#### Using non-ARP allocation procedures in platform trials in the absence of ARP alternatives

Zhao [76] reported using the mass weighted urn design (MWUD) [71] in a 10-arm trial with Bayesian adaptive randomization where the updates to the randomization ratio occurred every ten weeks. While the MWUD accurately targets any allocation (equal or unequal, 2-arm or multi-arm) without approximation, it is not an ARP procedure. When the treatment imbalance control threshold is small, the unconditional allocation probabilities may slightly fluctuate at the early stage of the allocation sequence.

Zhao [76] also reported using the minimal sufficient balance (MSB) method [77] in a 5-arm Bayesian trial using RAR with allocation updated after every 50 participants while controlling the imbalances in 3 binary baseline covariates among treatment arms. The MSB method is not an ARP procedure. It is an alternate to stratified randomization and minimization for baseline covariate imbalance control. Since Bayesian adaptive randomization typically involves allocation ratios that, when approximated with integers, result in large block sizes, using an unequal allocation covariate-adaptive procedure based on mapping or stratified PBR (ARP procedures) might not result in an allocation ratio sufficiently close to the target one in sets of 50 participants. The unpredictable small sizes of the strata defined by baseline covariates prohibit the use of stratified randomization for unequal allocations in a sample of a modest size.

In these examples the parameters of the procedures were chosen to reduce the variations in the unconditional allocation ratio – as would be recommended whenever non-ARP procedures are used.

### Flexibility to accommodate for varying eligibility and center restrictions in platform trials

In many platform trials, the eligibility criteria may differ among the treatment arms. This could be due to perceived treatment benefits in biomarker driven subpopulations, tolerability issues (e.g., renal impairment), or other reasons. For example, in the ACTIV-2 COVID-19 trial [78], individuals with inflammatory skin conditions were excluded from randomization to injectable agents, while those with severe liver or kidney disease were excluded from randomization to orally administered camostat. In any case, these differing eligibility criteria will impact the composition of the subpopulations in the remaining treatment arms of the trial.

A similar limitation to randomization may exist from the center perspective. Treatment arms are typically added to platform trials through the implementation of the so-called intervention specific appendices (ISAs) to the master protocol that describe the rationale and the procedures for an added intervention-specific subpopulation. Not all centers need to implement the ISA. As such, some treatment arms may not be open for randomization at some of the centers. One reason could be that the center or a whole region does not consider the novel intervention as appropriate and as such does not provide approval for the ISA.

Overall, randomization methods for platform trials should suitably handle “exclusion” of some treatment arms for randomization on a participant/center level, while still achieving the target allocation ratios in the trial. When a shared control arm is used for a platform trial, different intervention-specific eligibility criteria pose methodological challenges [23, 29, 45, 79]. For a multi-arm trial this might be accounted for by using the most stringent set of eligibility criteria – the intersection of all eligibility criteria – for all treatment arms in the trial. While this might introduce bias in selection of the population narrower than some of the interventions are intended to, it is typically not feasible for platform trials, as not all interventions – and thus their corresponding eligibility criteria – might be known in advance. Even if the platform trial does not start out with different eligibility criteria between the treatment arms, due to the open nature of platform trials, a future treatment arm might bring differing eligibility. Therefore, an appropriate randomization method dealing with varying eligibility criteria, for example, one of the methods described below, should be selected in the planning phase of a platform trial.

**Fig. 6:**
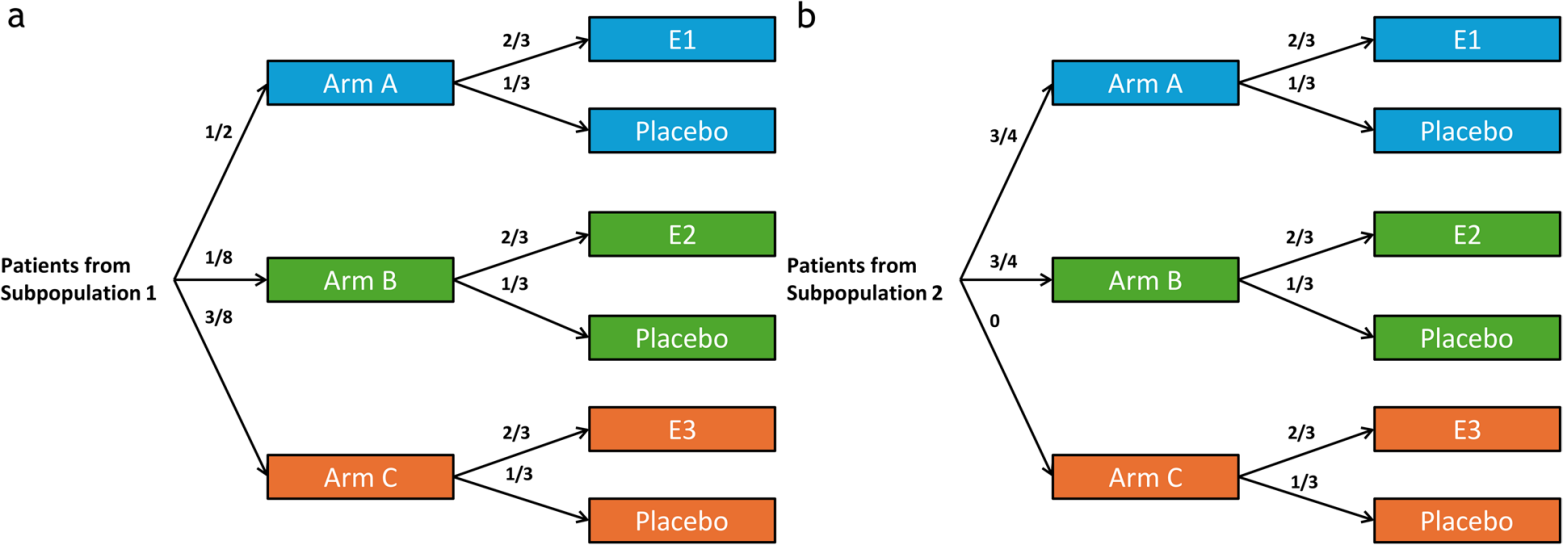
Randomization probabilities for participants in subpopulation 1 (eligible to all arms) and subpopulation 2 (eligible to Batch A and B only)

A possible approach to dealing with varying eligibility within the analysis is to restrict analysis to a distinct subset of the control data that aligns with the respective intervention-specific eligibility criteria. This was implemented in the ACTIV-2 COVID-19 trial mentioned earlier. In this trial, the pooled control consisted of all control patients, who were concurrently allocated and would have been eligible for the respective treatment arm. The trial employed a 2-step randomization method with varying allocation probabilities in both steps, depending on the number of treatment arms the next patient was eligible for. However, in such an approach, participants might be imbalanced with respect to the different eligibility subsets of the experimental arms between the control and interventions, which can lead to bias [45]. For illustration, consider a clinical trial with three experimental treatments (E1, E2, E3) and a control. All participants satisfy the inclusion/exclusion criteria of E1 and E2, but E3 is more restrictive, thus dividing the participants into two distinct subpopulations: those eligible for all treatments (subpopulation 1) and those only eligible for E1 and E2 (subpopulation 2). Now, consider a trial employing a 2-step randomization method, where participants from subpopulation 1 are assigned to Batch A (receiving E1 or matching control) with probability 1/2, to Batch B (receiving E2 or matching control) with probability 1/8, and to Batch C (receiving E3 or matching control) with probability 3/8, as illustrated in Fig. 6 (left plot). Participants from subpopulation 2 are allocated with a 6/8 probability to Batch A, and with a 2/8 probability to Batch B, as shown in Fig. 6 (right plot). Within each Batch, participants are randomized to experimental treatments vs. matching control in a 2:1 ratio.

Assuming 120 participants from subpopulation 1 and 24 participants from subpopulation 2 are enrolled in the trial, we would expect an average of 40 participants from subpopulation 1 and 8 participants from subpopulation 2 in the respective control data for treatment 1. Meanwhile, in E1, there would be, on average, 40 participants from subpopulation 1 and 12 participants from subpopulation 2, yielding control-to-treatment ratios of 1:1 and 1:1.5 for the respective subpopulations, as detailed in Table 3.

**Table 3:** Average number of participants in subpopulations 1 and 2 in control and intervention of batch A

	Expected number of control participants in Batch A	Expected number of interventional participants in Batch A	Ratio control: treatment
Subpopulation 1	$120\cdot(1/2 + 1/8 + 3/8)\cdot 1/3=40$	$120 \cdot 1/2 \cdot 2/3 = 40$	$1:1$
Subpopulation 2	$24 \cdot (3/4 + 1/4) \cdot 1/3 = 8$	$24 \cdot 3/4 \cdot 2/3 = 12$	$1:1.5$

This disproportionate allocation of participants from subpopulation 2 to the control of treatment 1 may introduce bias. For instance, if treatment 3 is more efficacious and designated for participants with stronger immune systems (i.e., subpopulation 1), then subpopulation 2, which includes participants ineligible for E3, may be, on average, less healthy and more likely to exhibit a less favorable outcome. Consequently, the outcome could be biased as a larger proportion of participants from subpopulation 1 receive E1 compared to the proportion of participants from subpopulation 2 who receive E1, when evaluated against the pooled control.

Viele [45] proposed a 2-step randomization method, which chooses the randomization probabilities based on user chosen (fixed) weights for each experimental treatment/matching control pair, such that, on average, the ratio of the respective subpopulations is balanced between the experimental and pooled control arms, while being able to deal with arms being opened and closed in the trial. If an ARP procedure is chosen to implement the randomization within each subpopulation, this method ensures the ARP property (for each subpopulation) but does not incorporate stratification and cannot deal with RAR.

Selukar et al. [80] suggested including the eligibility subpopulations as a stratification factor. However, when the number of experimental arms is high and/or other strata are present, this might be impractical due to the excessive number of resulting strata. Another approach proposed by Selukar et al. [80] is to extend a dynamic balancing [81, 82] or the minimization algorithm [55, 56]. In a dynamic balancing, an experimental arm that minimizes covariate imbalance relative to the respective control after hypothetically allocating the next participant, is assigned the highest probability for the next participant allocation. This imbalance is evaluated by using a chosen imbalance function, which Selukar et al. [80] recommended adapting for varying eligibility by separately calculating the imbalance for each experimental arm data and its corresponding control data with respect to the eligibility with the hypothetical allocation, as opposed to a single calculation for both the experimental arm data and control data with the hypothetical allocation. The utility of these methods in maintaining allocation ratios and achieving balance in stratification factors has not been explored. The authors pointed out that the efficiency of the platform trial decreases as the inclusion/exclusion criteria between the treatment arms diverge, due to the reduced sharing of control data. It is also important to acknowledge that dynamic balancing with an unbalanced allocation ratio introduces unique challenges [49, 52].

And finally, the randomization of participants with different eligibility criteria can be accomplished by already existing tools – e.g., by following the pre-generated randomization schedule and skipping the allocations a participant is not eligible to be later backfilled by eligible participants. If most of the skipped slots on the randomization schedule are immediately backfilled (which would be the case, for example, when only a small fraction of participants is not eligible to all treatment arms), the treatment arm totals at the end of randomization will be close to the target ones. While using this backfilling approach achieves closer to the target allocation ratio, consideration of its use should take into account cases with larger fractions of backfilling where it can lead to consecutive assignment of the same treatment arm. This option is likely to be considered when stratification by the eligibility subset leads to too many strata, however, the properties of this dynamic randomization schedule-guided method were not explored and need to be further explored in setting specific to the trial in question.

If randomization is stratified by eligibility criteria, then within each stratum the ARP property is preserved. Otherwise, the unconditional allocation ratio depends on the sequence of eligibility-defining covariates of the participants enrolled in the trial. The implications of this phenomenon need to be further explored.

### Randomization challenges related to limited drug supplies in multi-center platform trials

To accelerate drug development, most platform trials are conducted across multiple centers, often involving many small centers. In this context, stratifying randomization by center can create numerous small strata, which may reduce the balance of treatment assignments and compromise statistical efficiency. Additional stratification by prognostic factors aggravates the situation, leading to many stratification cells with just 1 or 2 participants and failing to provide balance in prognostic factors. Thus, a central randomization, where participants are randomized along a single randomization schedule regardless of their center is commonly employed in multi-center trials. To stratify central randomization, a separate randomization schedule is prepared for each stratum and participants from different centers are randomized along the schedule for the stratum they belong to. The downside of the central randomization is that the allocations at any given center become unpredictable and the drug supplies for all treatment arms should be present at every center at any moment to support central allocation.

In a multi-center, multi-treatment platform trial, keeping the drug supplies for the full set of treatment arms at all centers at any point of randomization might be impractical or not even feasible. The drug supplies situation is often aggravated by platform trials use early in drug development, when investigational interventions are scarce, and motivates further investigation of randomization methods to accommodate for economical use of drug supplies at the trial centers.

Two dynamic allocation approaches that support randomization with limited drug supplies are modified Zelen’s approach [57, 83] and allocation with partial drug supplies sent to centers described by Morrissey et al. [84]. For example, the modified Zelen’s approach can be useful in a trial with equal allocation to 5 treatment arms where centers are sent a block of 5 treatment kits (one for each treatment arm) which are used for the first 5 enrolled participants at the center, following the central randomization schedule whenever possible and skipping allocations already assigned at a center to later backfill them with participants from other centers. After the first block of 5 treatment kits is used at the center, the second block of 5 treatment kits is delivered for randomization at the center. Thus, if a center randomizes a small number of participants, for example, 3 participants, the second block of 5 treatment kits will not need to be delivered to the center.

An even more economical technique is an allocation with partial drug supplies sent to the centers described by Morrissey et al. [84] – for example, when there are 7 treatment arms, but most centers are not expected to randomize more than 3 participants. In this case the centers are initially sent 3 treatment kits each and after 3 participants are randomized, the re-supplies also arrive in sets of 3. Similar to the modified Zelen’s approach, participants are randomized to the first unused treatment assignment on the randomization schedule with the treatment kit available at their center; unfilled assignments are backfilled by the participants randomized at other centers. One area of application is trials for rare diseases. Many platform trials are designed for rare diseases, where they may involve hundreds of centers, each expected to enroll at most a handful of participants, but with a huge variability in the enrollment times. For instance, if, on average, one participant per year is expected from each center, it may still be necessary to be prepared for the situation that two participants are enrolled consecutively, so each center should have at least 2 treatment kits available at any given moment but may not be able to keep the treatment kits for all treatment arms.

Both methods were introduced for trials with equal allocation; the drugs for all treatment arms were assumed to have the same appearance or come in kits that cannot be disassembled to use their components for other treatment arms. Both methods could be stratified by factors other than center and thus provide balance in important prognostic factors as well as within centers (with incomplete blocks randomized in centers, a reasonable balance is expected with small sizes).

Kuznetsova and Tymofyeyev [49] expanded these techniques to ARP unequal allocation procedures, including increased complexity of drug distribution with partial drug supplies sent to the centers that would comply with an ARP property. These procedures can also be used under the multiple-dummy blinding in a $\:K$-arm trial where a participant in an intervention arm receives one intervention and $\:(K-1)$ controls matching in appearance other interventions and a participant in a control arm receives $\:K$ controls. However, the use of randomization with partial blinding, where a participant is randomized to an intervention-specific batch and within that batch, to an intervention or control arm, have not been studied with these procedures – either with equal or unequal allocation.

In platform trials allowing for many interventions to be opened in parallel, it may not be practical that every participant is randomized among all of them. It can create an unnecessary burden to participants and affect compliance in turn affecting ITT and per protocol analysis. The number of treatment arms faced by a participant may be decreased by their ineligibility or by not providing consent to some of the treatment arms. From the design point of view, the number of opened treatment arms may also be limited formally, e.g., when there are more than 3 treatment arms, the participant is given a randomization only among 3 treatment arms (which may be further decreased by ineligibility and/or informed consent). As centers may be assigned which 3 treatment arms they offer (at least for a certain period of time) to their enrolled participants, this may have practical benefits; for instance, it relaxes the strict requirement of availability of all treatment kit types at every center at any point of randomization, overwhelming staff training, unprocessable informed consent, etc.

## Operational considerations

### IRT randomization considerations

Due to the complexity of the randomization in platform trials, an IRT system, also known as Randomization and Trial Management System (RTSM), is utilized. Two key consideration areas for the IRT randomization implementation include how to approach adaptations and how to handle treatment arm eligibility.

#### Randomization adaptations

As mentioned above, platform trials often include adaptive design elements which need to be accounted for in the IRT’s randomization implementation. These adaptations include:

- Open/Close/Pause/Reopen Treatment Arms [1, 85, 86]: This classification of adaptations is typically required for planned interim analyses purposes. For instance, interim analyses may evaluate effectiveness where a treatment may be closed if ineffective. Interim analysis may also require a treatment arm to be paused for safety/toxicity evaluation and then either reopened or permanently closed based on results. Additionally, a treatment arm may be closed once the target number of participants is achieved or reopened if the target number is increased.
- Treatment Arm Additions [1, 85, 86]: In platform trials, newly identified treatment arms are commonly introduced through protocol amendments.
- Allocation Ratio Adjustments [1, 22, 85, 86]: The allocation ratio may require adjustments for reasons such as treatment arm additions/closures, interim analyses (based on effectiveness/performance), shared control arms, target numbers of participants, etc. Whenever adaptations incur an allocation ratio change, the FDA 2023 guidance [22] suggests accounting for the time periods of different allocation ratios in comparisons between drugs (e.g., stratifying by the time period). Clear identification of the randomization periods can be achieved through beginning a new randomization schedule or starting with a new block (if using blocked randomization) when the ratio changes.

When designing the IRT’s randomization, how these adaptations are handled must be considered. Approaches for handling adaptations include:

- Real-time Adaptations: The IRT can be configured with the ability to open/close/pause/reopen treatment arms, add treatment arms, perform allocation ratio adjustments within an IRT user interface (UI). A designated user can enter treatment arm settings, which would then be applied to an adaptable randomization schedule in real-time. For instance, treatment arms set to open in the UI are included within the randomization schedule with their entered ratio weights and any treatment arms set to closed are excluded from the randomization schedule. This approach would not require amendments or new randomization schedules for any adaptations that are entered.
- System Amendments: The initial IRT system is set up with predefined randomization schedules that include the treatment arms and allocation ratio specified in the initial version of the protocol. Any adaptation would require an IRT amendment with the inclusion of a new randomization schedule.
- Hybrid (Combination of Selected Real-Time Adaptations and System Amendments): The IRT can be set up to perform certain adaptations in real time and handle other adaptations through system amendments. For instance, the IRT can include predefined randomization schedules and the IRT UI configured with the ability to open and close treatment arms (only). If a new treatment arm was introduced through a protocol amendment and/or if there were a ratio adjustment required, the IRT would undergo a system amendment to add the new randomization schedule with the additional treatment arm and/or adjusted allocation ratio.

Ultimately, the randomization of most platform trials has some element of real-time adaptations, but this is particularly driven by timelines. The extent of real-time randomization adaptations that are included within the IRT functionality depends on the randomization method (e.g., fixed randomization, blocked randomization, ARP procedure, RAR), expected adaptations, the initial timelines, amendment timelines, sponsor/trial team preferences, etc.

#### Treatment arm eligibility

**Fig. 7:**
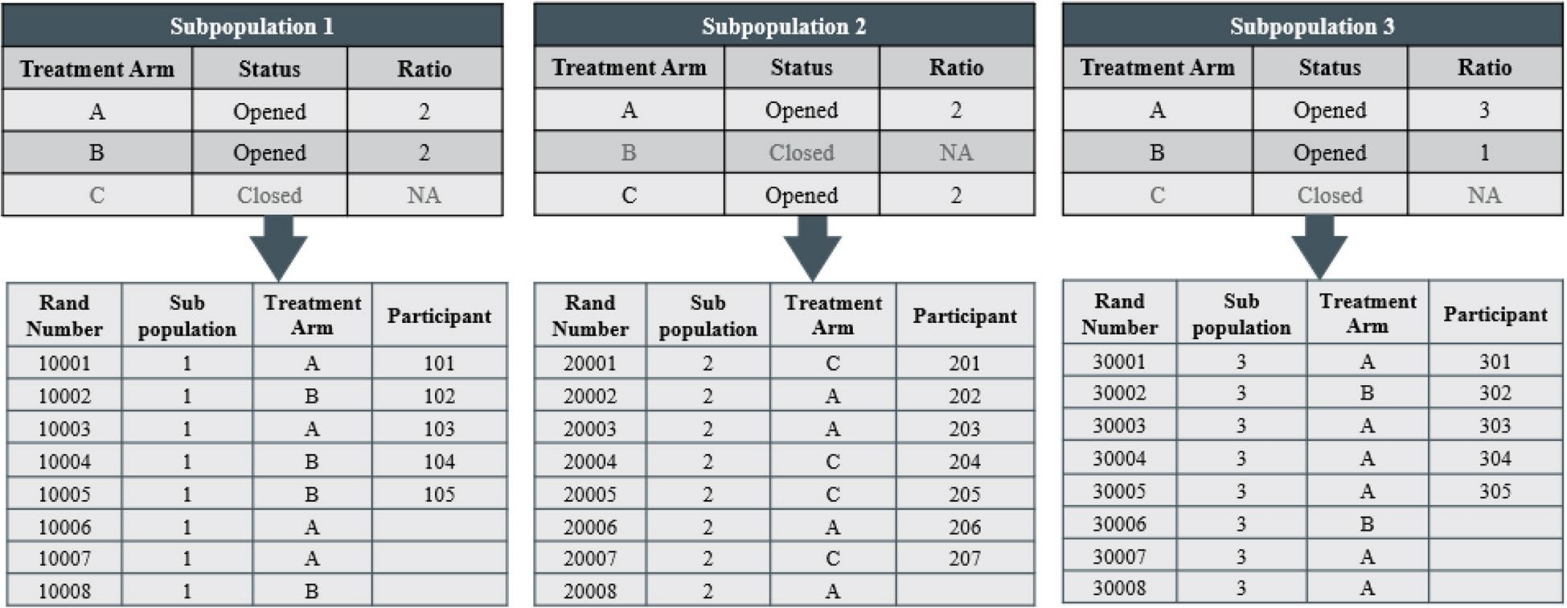
An example of the adaptation management IRT UI and randomization concept for three independent subpopulations (1, 2, and 3) in a platform trial. Treatment arms A, B, and C are involved in the platform trial, but status and ratio can differ across each subpopulation as below. Participants are assigned within the randomization schedule associated with their subpopulation and assigned to the next treatment within the randomization schedule. For instance, participants belonging to subpopulation 1 are ineligible for treatment arm C. Thus, in the IRT UI, treatment arm C is set to closed and that treatment arm is excluded from the corresponding randomization schedule. Five participants are assigned within subpopulation 1 to either treatment arms A or B

**Fig. 8:**
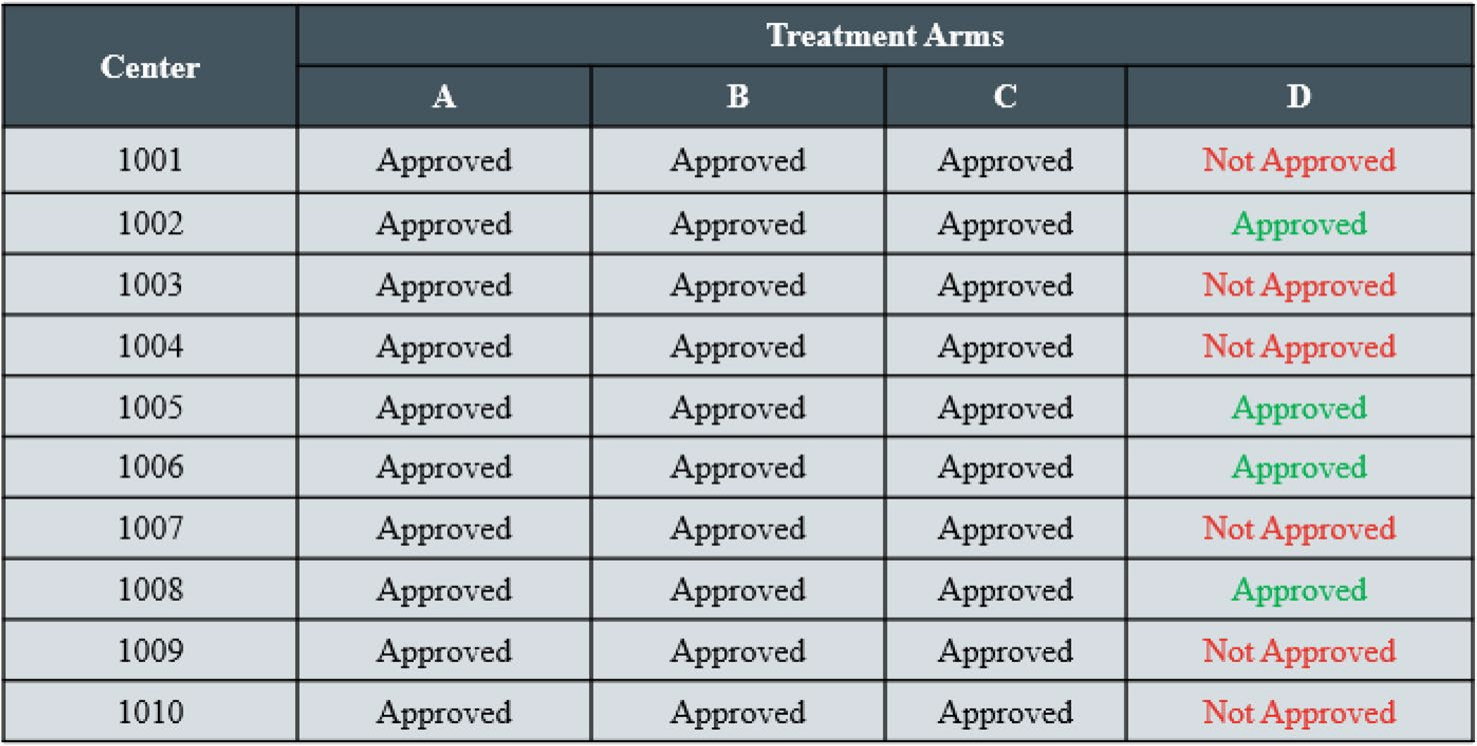
A conceptual example of how eligibility for individual centers in a platform trial could be managed within an IRT UI

As noted within the FDA 2023 guidance [22], master protocols may utilize treatment arm specific eligibility criteria. When master protocols require this eligibility criteria, the guidance [22] instructs that randomization processes should be designed to prevent participants from being randomized to treatment arms for which they are ineligible. There are different ways that eligibility can vary and be handled by the randomization implementation in the IRT which include:

- Eligibility by Subpopulation: Treatment arm eligibility can impact subpopulations of participants, where each subpopulation contains unique treatment arm inclusions and exclusions for randomization. These subpopulations may be classified based on elements such as stratification, subpopulation characteristics/specific eligibility criteria, region, etc. For instance, a trial may include treatment arms that target specific biomarkers where the subpopulations are defined based on specific biomarker presence. Another example is where a trial may include different Standard of Care (SOC) treatment control arms that differ across regions, where subpopulations are defined based on the SOC specific regions. These subpopulations with differing treatment arm eligibility will each require an independent randomization schedule and adaptation management (e.g., IRT UI if performing real-time adaptations), and IRT logic for subpopulation classification/assignment in appropriate randomization schedule. Fig. 7 shows a simple example of the treatment arm adaptation management IRT UI and randomization concept for three independent subpopulations. This is an approach to managing eligibility at the subpopulation level (e.g., if a treatment arm is closed for a subpopulation, then that treatment arm is excluded from that subpopulation’s randomization schedule in the IRT). The subpopulation definitions, ratio parameters (e.g., ratio in whole numbers, ratio weight within blocks, probabilities) and the randomization method (e.g., schedule/structure, algorithm/probabilistic assignment) in practice would be designed based on the randomization method specified in the platform trial’s protocol and the decisions of trial stakeholders (e.g., statisticians, clinical investigators, trial managers).

- Eligibility by Individual Participant: If the protocol allows for individual eligibility (outside of subpopulations), then the IRT’s randomization can collect eligibility criteria for each participant and prevent assignment to any ineligible treatment arms (e.g., by algorithm logic, through skipping records within randomization schedule, or assignment in a separate schedule).
- Eligibility by Center: Center eligibility can vary when new treatment arms are added. Prior to initiating randomization to a newly opened treatment arm, approval from a center’s Institutional Review Board (IRB) is typically required. Some centers may never be approved due to regional regulations, while the timing of IRB approval may vary for others. Additionally, center-readiness may differ in timing for the initial treatment kit shipment. Including center-level eligibility controls is a common requirement for IRT functionality in platform trials. Fig. 8 provides a conceptual example of how eligibility for individual centers could be managed within an IRT UI for when a treatment arm (in the example treatment arm D) is added to the platform that has different center-level eligibility. The IRT UI can also be configured to batch approve centers at the regional level. If a center is not approved for a treatment, then randomization to that treatment arm is prevented at that center. 

### IRT medication management considerations

Medication management in platform trials can also involve complexity, which requires an IRT system to be utilized. Two main consideration areas for the IRT medication management implementation include how to handle the addition of new treatment kit types and the maintenance of blinding for treatment kit types.

#### New treatment kit types

Adding a new treatment arm often brings the complexity of adding of its corresponding treatment kit type to the trial. The adaptation/amendment strategy should also consider adding potential new treatment kit types in the IRT design. The level of configurable real-time adaptations depends on what is known about future treatment kit types. For instance, if future treatment kit types are expected to follow existing dispensing schedules, use different volumes or combinations of existing treatment kit types, some real-time adaptations may be possible within an IRT UI. However, for many platform designs, the characteristics of the new treatment kit types are typically unknown at the time of the initial IRT build. In these cases, any new treatment kit type would require an IRT amendment. Regardless of the IRT approach, there usually are other actions required from the medication management perspective, such as creation of new kit schedule (if using numbered supplies), packaging, labeling, shipping to centers, etc. Furthermore, clinical supply management within adaptive designs can pose challenges due to uncertainty and complexity [87]. Since platform trials are master protocols with adaptive designs, they typically bring in even more uncertainties and complexities around the timing and nature of adding new treatment arms (e.g., number of treatment kit types can be high, possibility of multiple different sources). Thus, during the planning phase, all aspects of clinical supply management (both related to the IRT design and outside of the IRT) should be carefully considered.

#### Blinding considerations for treatment kit types

As treatment kit types of different dispensing appearance are added to a platform trial, unblinding concerns become highly prevalent. Thus, when introducing new treatment kit types, unblinding risks should be carefully evaluated. Kit types may have noticeable differences such as appearances, routes of administrations, dosing schedules, etc. FDA [22] presents some blinding strategies such as different uses of intervention-specific controls (e.g., multiple-dummy, double-dummy), and recommends that sponsors discuss their proposed approach with regulators early during planning. However, the use of intervention-specific controls may not be feasible since it could be costly and burdensome to participants, especially in platform trials where there are several interventions. If these differences are not mitigated by intervention-specific controls and blinding is still required, they likely will be observed by blinded IRT users.

Unblinding risks can also stem from treatment kit types being sourced from different sponsors. Case in point, one treatment kit type is provided by sponsor A, while another is provided by sponsor B, and each produces independent kit schedules. When kit schedules originate from different sponsors, they will likely have different kit number ranges. In a double-blind trial, if kit numbers are visible to blinded trial personnel, they may notice differences in numbering. This can be partially unblinding with possible identification of participants on different treatment arms. A similar problem occurs where some treatment kit types are numbered, while others are non-numbered (locally sourced or handled as bulk supply). The presence or absence of kit numbers can be partially unblinding.

These differences may not be handled through intervention-specific controls or packaging/labeling strategies for various reasons or limitations, and they need to be addressed in other ways. Other recommendations for alleviating these unblinding risks may include utilizing unblinded pharmacist roles, and to ensure that the kit numbers are not visible to blinded trial personnel within the IRT or other systems (excluding blinded reports, data transfers, confirmations, etc.)

Additional opportunity for partial unblinding arises when a new treatment arm with a new treatment kit type is added to a platform trial and the way trial centers handle treatment kits from prior shipments. Consider a trial center that keeps the treatment kits received prior to the new treatment arm addition (old treatment kits) separate from treatment kits received in a shipment shortly after the new treatment arm addition (new treatment kits). Center staff may assume that new shipment likely includes the treatment kit type for the new treatment arm. In this case, if a participant receives an old treatment kit at randomization, the center staff may know that participant was not assigned to the new treatment arm (a partial unblinding). If treatment kits are dispensed at multiple visits in the trial, the IRT can use the old treatment kits only for the non-randomization visits of the previously randomized participants to avoid a partial unblinding of this kind.

It is important to note that there are trials where blinding is paramount and others where this may be less so [88]. For instance, blinding may be less of a priority in early phase platform trials than in platform trials with a confirmatory component. There is also the question of how feasible blinding is in a trial. This is caveated by the type of intervention being investigated and how effectively these interventions can be blinded or dummy blinded. For example, there may be situations of relevance for platform trials, where blinding generally cannot be implemented (e.g., in oncology), or where reactions to the drug may reveal treatment assignment (e.g., color of urine for some tuberculosis treatments). There are also cases where all interventions in a platform trial have different appearances. For these cases, utilizing a double-dummy approach (i.e., introduction of intervention-specific control for each intervention) may require participants to take an abundance of treatment kits (mostly controls). This approach effectively maintains the blind, but it is extremely burdensome to trial participants. If the level of blinding cannot effectively be implemented as double blind, additional analysis considerations which can considerably impact the target allocation ratio at design and need to be addressed in the statistical analysis plan [88].

### Establishing operational strategy plan for adding treatment arms

Compared to traditional parallel-group trials, master protocol writing (i.e., platform trial planning) should begin earlier to account for stakeholder coordination, infrastructure requirements, and complex trial design elements [1, 86, 89]. This planning process should pay particular attention at ensuring the effective coordination and execution of adding treatment arms is well covered. This plan can be established after the IRT design is determined (as well as other applicable processes/systems) for the platform trial. Since it may be some time before a new treatment arm is added, it is important to establish this plan upfront during the start-up phase when these important design components and systems are familiar.

#### Derive list of required activities

When a new treatment arm is identified to be added to the platform, there are several activities required before it can be incorporated into the IRT. Some examples of required activities include committee meetings, protocol revisions (e.g., addition of treatment arm/treatment kit type/visit schedule to the master protocol, addition of new intervention-specific appendix), regulatory and IRB protocol review/approval, treatment kit manufacturing/packaging/labeling/shipping), electronic data capture (EDC) updates/amendment, new consent forms, IRT amendments (e.g., add treatment arms, treatment kit type, or visit schedule), IRT real-time adaptation configuration UI updates (e.g., center approvals, open treatment arm(s), set allocation ratio).

Since there are many tasks required when adding a new treatment arm, these need to be well-planned and coordinated for successful execution. These activities may have different roles/stakeholders, timelines, and level of effort. This plan should cover each activity required, along with individuals/parties responsible for each activity, the level of effort, timelines, etc.

#### Designating roles and responsibilities

Designating individuals, parties, or responsible roles is an important step in the planning phase. For instance, if the IRT has an adaptation UI, it should be determined which role(s)/individual(s) will be responsible for performing the adaptations. This usually depends on what parameters are included within the UI and the relevant area of expertise required. If the UI includes parameter settings that require statistical evaluation/decision making, then it is recommended that a statistician either be responsible or consulted for any updates. If there are multiple areas of expertise required, then representatives in each area should collaborate prior to performing updates.

After the designated roles are determined, it is recommended to put guidance materials (e.g., quick reference guides, working instructions, mandatory training presentations) in place. Since platform trials can be much longer than a traditional trial, where there may be lengthy periods of time between adaptations and potential staff turnover, these guidance materials will be helpful in ensuring these tasks are accurately executed.

#### Timelines

For the process of adding a new treatment arm, timing is an important consideration (i.e., when to initiate each activity based on how long each takes, when each activity needs to be completed). Establishing a timeline into the plan is recommended (i.e., when each role/party needs to be contacted to begin work and when they need to start/finish). To figure out the overall time allotted for the process, which would begin when a new treatment arm is identified and ends with target time for first participant randomized to the treatment arm. Then, working within this overall time frame, it can be determined time to initiate as well as timelines and deadlines for each task.

A main IRT related consideration for timing includes when to initiate the IRT vendor to begin work for the amendment based on how long it is expected to take from start to finish. This amount of time depends on the scope (e.g., level of real-time adaptations incorporated vs. system functionality amendments required, if user acceptance tests (UAT) are required). This also depends on the fact if the change is within the boundary of what was planned initially or if it otherwise included as additional unforeseen changes (e.g., add new dosing calculation, add new randomization method). Other IRT elements for timelines include when any user required actions are to be completed (e.g., medication management activities, center approvals, UI configurations).

### Operational considerations: summary

While many of the examples above are specific to IRT, this plan should also cover all activities required outside of the IRT (e.g., treatment kit packaging/labeling/shipping, IRB review/approval, center readiness activities) to ensure accurate and efficient execution of introduction of new treatment arms. Once the plan is established, tools such as worksheets or other planning applications to track each task will be helpful. The tracking tool can include useful information such as listing of each activity, date calculations to assist with initiation and due dates, roles/parties responsible and their contact information, etc. Deriving this plan may take sufficient effort upfront; however, having a well-thought-out amendment plan will ensure efficiency for the life of the platform trial. This is important since, as mentioned above, the success of platform trials requires the ability to efficiently add new treatment arms with minimal disruption as often as needed.

## Conclusions

Platform trials present numerous randomization challenges that are almost always not properly discussed at the design stage. Some of these challenges – such as the (more frequent) changes in the allocation ratio, the need to closely match the target allocation ratio (possibly given by an irrational number), the need for managing different eligibility criteria for the treatment arms – are shared with umbrella trials or trials with RAR. However, the mere addition and removal of the treatment arms in platform trials brings a sufficiently high level of complexity to deliver the randomization component of platform trials.

We have focused on some of the challenges, but there are many more that did not make it into this paper. While novel approaches are being developed to meet the randomization requirements of platform trials, we want to raise awareness of the importance of preserving the unconditional allocation ratio for participants allocated first, second, and so on. A failure to preserve the unconditional allocation ratio at every allocation may lead to the selection and evaluation bias even in a double-blind trial, accidental bias (especially when randomization is stratified by center), and reduced power of the re-randomization test [50, 52]. Some ARP unequal allocation procedures are described in this paper, but in other cases the ARP solutions do not exist yet. One example is the covariate-adaptive allocation for a multi-arm trial with RAR of the sets of participants. If the allocation ratio derived for the next set is represented by large integers, covariate-adaptive procedures expanded to unequal allocation through mapping will not provide a satisfactory solution. In this case one might have to use a non-ARP approach and try to choose the procedure that minimizes the variations in the unconditional allocations ratio to reduce the problems; also, operational measures could be taken to reduce the potential for bias.

Very technically advanced methods have been developed to derive optimal allocation ratios for sets of participants in trials with RAR. However, when the randomization is implemented using the complete randomization or permuted block randomization that approximates the target allocation ratio with large integers, the achieved allocation ratio might substantially differ from the desired one, considerably impacting the performance characteristics of the design. The issue is aggravated by the fact that the exact size of the randomized of participants set is often unknown in advance – for example, when data are evaluated on a monthly basis. In this case, the brick tunnel randomization [48] can help approximate the target allocation ratio closely even in small sets of arbitrary size. It can also help to randomize the treatment arms in unconventional allocation ratio that arises in non-response-adaptive trials from efficiency considerations or the need to add a treatment arm to randomization.

Randomization methods that account for different eligibility criteria across treatment arms represent a new class of procedures, not described in other settings. Here, instead of having all participants randomized in the same allocation ratio, participants are randomized in a ratio dependent on their eligibility. Stratification by eligibility subpopulation offered by Selukar et al. [80] places the procedure in a familiar framework of stratified randomization, albeit with different allocation ratios across strata; it can easily handle unequal allocation ratios. The dynamic randomization that favors the treatment assignment that would result in the lowest imbalance between the treatment arm the participant is eligible to and the subset of control with the same eligibility criteria is very interesting. It differs from approaches without eligibility restrictions that typically consider imbalances across all treatment arms, not just the pairwise imbalances with the control. The properties and performance characteristics of such an approach should be further explored.

The feasibility of implementing complex randomization methods in a platform trial is a prominent consideration when choosing the randomization method for the trial. With multiple treatment arms that open and close, changes to the allocation ratio, differences in eligibility criteria, and blinding requirements, it is important to have a good plan and proper tools to support a fit for purpose execution of the randomization. The paper provides extensive recommendations regarding the IRT solutions that allow us to overcome the operational challenges caused by complex randomization and suggests areas where further work is much needed.

## Data Availability

All results reported in this paper are based either on theoretical considerations or simulation evidence.
